# Anthropogenic emissions from South Asia reverses the aerosol indirect effect over the northern Indian Ocean

**DOI:** 10.1038/s41598-020-74897-x

**Published:** 2020-10-27

**Authors:** Subin Jose, Vijayakumar S. Nair, S. Suresh Babu

**Affiliations:** Space Physics Laboratory, Vikram Sarabhai Space Centre, Trivandrum, India

**Keywords:** Climate sciences, Atmospheric science

## Abstract

Atmospheric aerosols play an important role in the formation of warm clouds by acting as efficient cloud condensation nuclei (CCN) and their interactions are believed to cool the Earth-Atmosphere system (‘first indirect effect or Twomey effect’) in a highly uncertain manner compared to the other forcing agents. Here we demonstrate using long-term (2003–2016) satellite observations (NASA’s A-train satellite constellations) over the northern Indian Ocean, that enhanced aerosol loading (due to anthropogenic emissions) can reverse the first indirect effect significantly. In contrast to Twomey effect, a statistically significant increase in cloud effective radius (CER, µm) is observed with respect to an increase in aerosol loading for clouds having low liquid water path (LWP < 75 g m^−2^) and drier cloud tops. Probable physical mechanisms for this effect are the intense competition for available water vapour due to higher concentrations of anthropogenic aerosols and entrainment of dry air on cloud tops. For such clouds, cloud water content showed a negative response to cloud droplet number concentrations and the estimated intrinsic radiative effect suggest a warming at the Top of the Atmosphere. Although uncertainties exist in quantifying aerosol-cloud interactions (ACI) using satellite observations, present study indicates the physical existence of anti-Twomey effect over the northern Indian Ocean during south Asian outflow.

## Introduction

In case of warm clouds having a fixed liquid water path, an increase in aerosol (which acts like cloud condensation nuclei (CCN)) concentrations leads to a decrease in cloud effective radius (CER) and an enhancement in cloud albedo. This is usually referred as “first indirect effect” or “Twomey effect”^1^, and ship track emissions studies^2,3^ have provided one of the well-known observational evidence of this effect. The smaller the cloud drops, the weaker drop coalescences leading to suppression of precipitation and enhancement in cloud lifetime^4^ (“second indirect effect”). However, modelling and observational studies have found evidences that both support and disagree with this lifetime hypothesis^5,6^. Enhancement in CCN concentrations can also lead to thinning of cloud layers by decreasing (increasing) sedimentation (entrainment) rate, and such effects are found to be prominent if cloud base heights are greater than 400 m^7^. Suppression of warm rain due to enhanced aerosol loading can elevate smaller droplets under convective conditions. Freezing of large number smaller droplets at higher altitudes release more latent heat and leads to the invigoration of convective clouds. This phenomenon (aerosol induced invigoration of convective clouds) is more prominent during weak wind shear conditions and for clouds with warm cloud base^8^. In addition to these effects, presence of absorbing aerosols embedded within or below (above) the cloud layer leads to reduction (enhancement) in cloudiness and such effects are referred as rapid adjustment or “semi-direct effect”^9^. The mean effective radiative forcing associated with aerosol-cloud interactions is found to be − 0.55 W m^–2^ and with low confidence^10^.

Among the different cloud types, aerosol mediated changes in warm cloud properties are extensively studied, yet their estimated radiative effects are still highly uncertain^10^. This high uncertainty stems from our inability to disentangle the effect of meteorology from the aerosol impact on cloud microphysics^11^. Theoretical^5^ and observational studies^12^ revealed that aerosol induced changes in macro and microphysical properties of warm clouds are determined by the competition between moistening of cloud layers by precipitation suppression and drying by enhanced entrainment of overlying air. Cloud brightening is prominent if the overlying air is moist and a reverse effect can occur in case of mixing with dry free tropospheric air^13^. Recent study^14^ on cloud liquid water path (LWP) response to cloud droplet number concentrations (N_d_) revealed that aerosol response to cloud LWP modifications strongly depends on the prevailing relative humidity of the region. Over highly polluted regions (like ship tracks, oil refineries, wildfires etc.) a weak average decrease in cloud water content is observed with respect to aerosol loading which induces an offset of ~ 23% in cooling due to Twomey effect^15^. In addition, few studies conducted over highly polluted regions^16,17^ across the globe also reported the role of aerosols in counter-intuitive cloud microphysical changes (increase in cloud effective radius with aerosol loading).

On the backdrop of these recent observational evidences it will be interesting to study aerosol-cloud interactions (ACI) over the northern Indian Ocean especially during winter months (December–February). During these months, transport of dry air mass laden with pollutants from South Asia makes it one of the aerosol hotspot regions in the globe^18^. Radiative effects of these pollutants on the regional energy balance was well documented during INDOEX (Indian Ocean Experiment) and reported that the first indirect effect almost compensates the warming induced by the direct effect of clouds^18^. Several shipborne and aircraft based thematic campaigns (eg. ICARB^19^, ARMEX^20^, BOBEX^21^, CARDEX^22^ etc.) carried out over the northern Indian Ocean after INDOEX also revealed the complexity and absorbing nature of South Asian outflows. Long term trend in aerosol loading over South Asia using in-situ observations explicitly revealed the significant increase in anthropogenic emissions (~ 4% increase in aerosol optical depth per year)^23^ during the recent decades. Here, we demonstrate the effects of significant increase in aerosol loading on the properties of warm clouds over the oceanic regions lying in the downwind of South Asian outflow.

## Results and discussions

**Figure 1:**
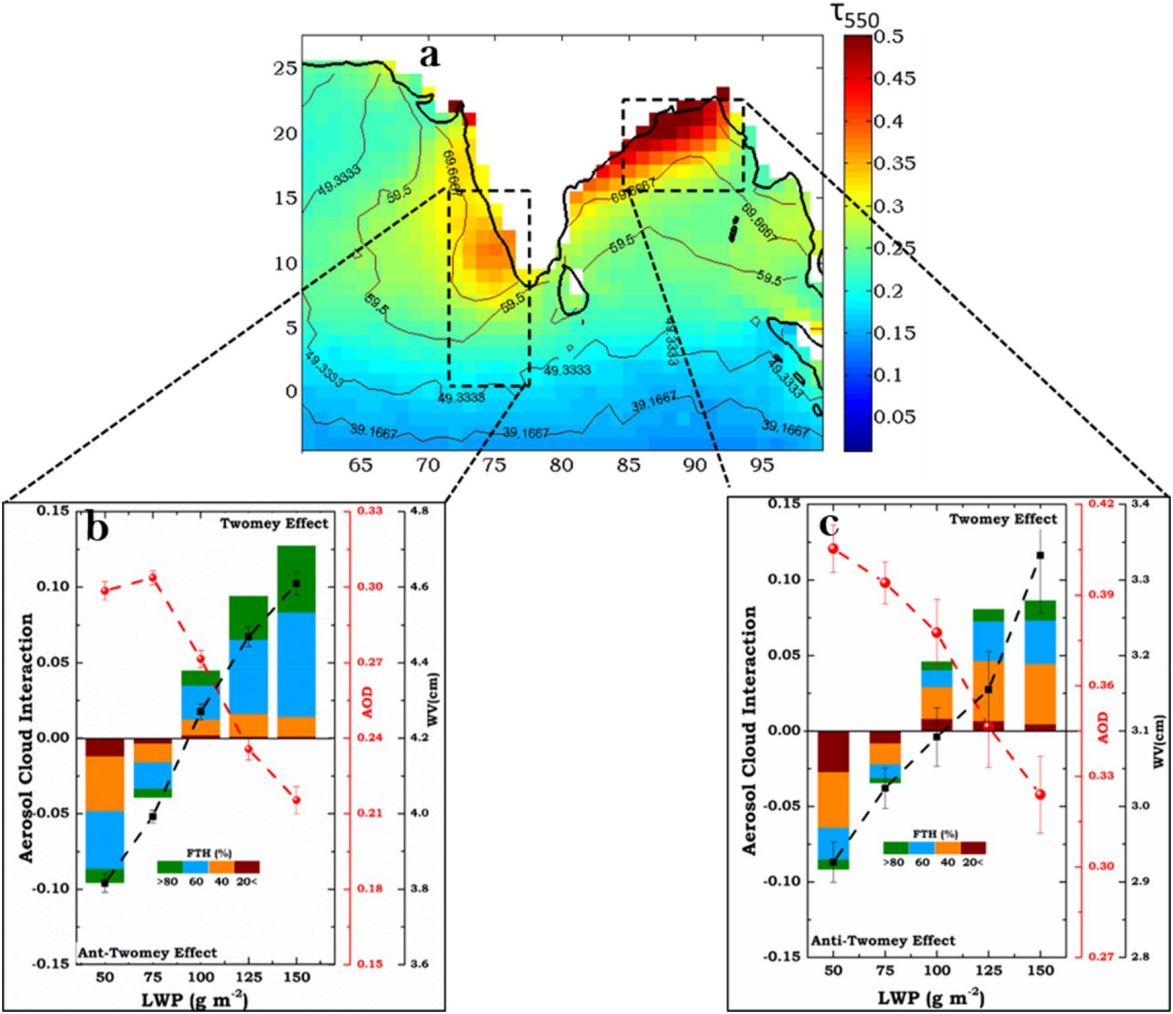
(**a**) Long-term (2003–2017) aerosol loading (AOD_550_) over South Asia derived from MODIS sensor onboard Aqua satellite. Contour indicates the anthropogenic fraction to total AOD (%). Rectangular boxes represent the study area SA1 and SA2 respectively. (**b**,**c**) Aerosol cloud interaction over SA1 and SA2 respectively for different cloud liquid water path (LWP) bins. Red and black curve in (**b**,**c**) represent AOD_550_ and precipitable water vapour (PWV, cm) corresponding to respective cloud LWP bin used in the estimation of ACI. Colorbars in (**b**,**c**) represent the percentage occurrence of free tropospheric humidity (FTH_700hPa_, %).

Spatial distribution of long term (2003–2017) mean aerosol optical depth at 550 nm (AOD_550,_ retrieved from Aqua-MODIS satellite) over the northern Indian Ocean during winter months shows high aerosol loading (AOD > 0.3) over the continental outflow regions (Fig. 1a). Analysis revealed that anthropogenic sources contributed ~ 60–70% to this high aerosol loading and long term satellite-based trend in AOD_550_ showed a significant increase (~ 2.2–2.3% year^−1^) over the northern Indian Ocean. For quantifying ACI, we considered two aerosol hot spot regions one over the Arabian Sea and the other over the Bay of Bengal respectively (now onwards study area, SA1 and SA2 respectively). Long-term mean AOD_550_ over SA1 and SA2 is found to be 0.28 ± 0.06 and 0.38 ± 0.08 respectively and both regions showed a statistically significant positive AOD trend (~ 0.1 AOD decade^−1^, supplementary Figure-01 and supplementary table-01). Vertical distribution of aerosol loading observed over the study area (supplementary Figure-02) using space-borne LIDAR revealed that majority of aerosols are confined in the lower atmosphere up to 2–3 km from mean sea level. Percentage occurrence of clouds over the study region estimated from MODIS data showed that ~ 40–60% of the cloud cover during dry months are mostly low-level clouds (700 hPa < Cloud Top Pressure (CTP) < 980 hPa) and a majority of them (greater than 75%) are having low LWP (< 75 g m^−2^; supplementary Figure-03). Above observations indicate the high possibility of aerosol interactions on marine warm clouds over the study regions.

ACI on marine warm clouds for different cloud liquid water paths (height of the bar indicates the value of ACI) over SA1 and SA2 is estimated (see “Methods”) and depicted in Fig. 1b,c. Decrease in CER with aerosol loading (Twomey effect) is observed for clouds with liquid water path greater than 75 g m^−2^ (hereafter high LWP regime); while a statistically significant increase in CER (supplementary Figure-04, supplementary Table-02) with the increase in aerosol loading (anti-Twomey effect) is found for clouds having low LWP (LWP < 75 g m^−2^). Over the northern Indian Ocean, previous studies (supplementary Table-03) using satellite observations have reported both Twomey as well as anti-Twomey effects. Aircraft based observations^24^ on cloud microphysics properties over the northern Indian Ocean during winter months reported ~ 35% decrease in CER for polluted clouds in comparison with pristine; while using satellite observations a weak positive correlation (r = 0.12) is observed between cloud-droplet size and AOD^25^. However, previous studies were confined to a specific LWP range and hence able to observe the contrast in ACI between high and low LWP clouds. Similar anti-Twomey effect is also reported from extremely high aerosol loading regions around the globe (Hebei, China^26^, Yangtze River Delta^27^, southeastern China^28^ and over industrial regions in Europe, China and North America^16^).

Modelling and observational studies^29,30^ suggested the possibility of anti-Twomey effect in warm clouds under the enhanced presence of large hygroscopic aerosol particles. Such particles hinder the growth of smaller particles (eventually leading to its evaporation) by suppression of supersaturation at the early stages of their activation. Over the northern Indian Ocean enhanced aerosol loading during winter months is mostly due to the presence of fine mode anthropogenic particles (Figs. 1 and Supplementary Figure-03) with major contributions from non-sea salt sulphate (nss-${\text{SO}}_{4}^{2-}$) and carbonaceous aerosols^31^. Hygroscopicity (κ_s_) of nss-${\text{SO}}_{4}^{2-}$ is found to be 8–15% lower than pure sodium chloride^32^, while in the case of carbonaceous particles κ_s_ depends on the nature of mixing between organic and inorganic species^33^. In this study, we report anti-Twomey effects in low LWP clouds over the northern Indian Ocean under certain environmental conditions (also refer supplementary Figure-05). A comparison with high LWP clouds revealed that enhanced loading of anthropogenic aerosols and reduced moisture content (AOD is increased by ~ 20–25% and PWV showed a reduction of about ~ 7–14%) occurred in conjunction with low LWP clouds. Our observations suggest that role of intense competition for moisture by the enhanced presence of anthropogenic aerosols can be a probable mechanism for anti-Twomey effect over the study region.

In perfectly adiabatic liquid clouds, CER increases with height initially by diffusional growth followed by collision-coalescence processes^34^. Previous studies have demonstrated that entrainment mixing can also significantly affect cloud microphysics^35,36^. In this study, we used variations in lower tropospheric stability (LTS, K) and free troposphere humidity (FTH, %) to represent this physical process (refer “Methods”). During the study period over northern Indian Ocean atmospheric conditions were mostly stable with an average LTS of 15 ± 0.3 K (LTS for different LWP bin is shown in supplementary Table-02). Percentage occurrence of FTH for different LWP bins is represented by colorbars in Fig. 1b,c. Analysis revealed that anti-Twomey effect is dominant under stable and dry atmospheric conditions over the northern Indian Ocean. Stable layer effectively blocks the transfer of moisture from surface to the free troposphere; such that surface flux–driven turbulence drive the dynamics of the region below the inversion layer and those regions above are dominated by radiatively driven convection^37^. Presence of dry air above low-level clouds can deepen the marine boundary layer (MBL), which further intensifies cloud-top radiative cooling^38^. Over the northern Indian Ocean, we observed ~ 18–40 m increase in MBL (supplementary Figure-06) when low entraining and radiating humidity^12^ conditions persisted above cloud tops. Modelling and observational studies revealed that entrainment of dry air on stratocumulus (Sc) cloud tops can catalyse its transition to cumulus (Cu) clouds^38,39^ and if the type of mixing is extreme-inhomogeneous (time scale of evaporation is lesser than homogenisation following entrainment of dry air) it can result in a slight increase in CER during such transition^35^^.^

One of the major drawbacks of using passive satellite measurements for estimating ACI is that the satellite-retrieved aerosol products are mostly columnar and may come from aerosol layers other than that actually interact with cloud base. To overcome this artefact, we used physically interacting^40^ aerosol and cloud layers (aerosol and cloud layers are separated by a distance of ~ 100 m) by making use of concurrent observations from Cloud-Aerosol Lidar and Infrared Pathfinder Satellite Observations (CALIPSO) and MODIS (refer “Methods”). Variation of CER with aerosol index (AI) for physical interacting clouds also indicates the occurrence of anti-Twomey effect in cases of low LWP clouds and Twomey effect for high LWP clouds as discussed earlier (supplementary Figure-07b) which further strengthens the validity of our observation. Similar observations on anti-Twomey effect in low LWP clouds was also confirmed using ground-based and airborne observations^41^ over South Asia suggesting the possible physical mechanism rather than satellite retrieval errors.

**Figure 2:**
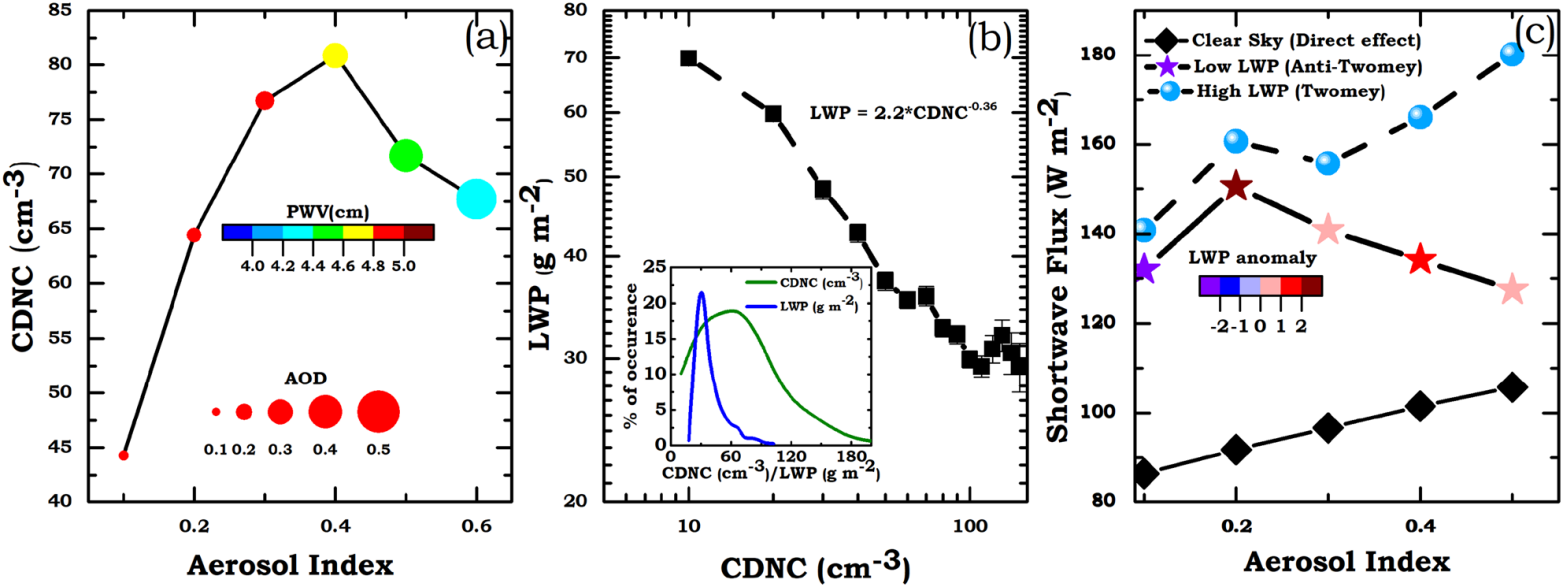
(**a**) CDNC as a function of AI, colorbar indicates precipitable water vapour (cm) at each AI bin and size of the bubble indicates respective AOD. (**b**) Variation of LWP as a function of CDNC (log–log scale). Inset shows the percentage occurrence of CDNC and LWP over the northern Indian Ocean. (**c**) Variation of Short wave flux with AI under clear sky (black diamonds) condition, for low (star, colorbar indicates LWP anomaly) and high (blue circles) LWP clouds.

Changes in CCN concentrations significantly affect the cloud droplet concentration (CDNC, N_d_) especially under low updraft conditions. It is observed that under low CCN concentrations, CDNC increases steeply, however the presence of bigger cloud drops suppress the activation of additional aerosol particles^42^. To study the variation of N_d_ (estimation of N_d_ is described in “Methods”) with CCN concentration, we used quasi coincident AI data (averaged within 50 km radius of N_d_ retrieval) and is shown in Fig. 2a. Our analysis revealed a non-linear increase (decrease) in N_d_ with AI at lower (higher) values of AI. Such negative relationships between N_d_ and AI are previously reported over land regions^43^. This observation (Fig. 2a) supports the role of enhanced aerosol concentrations leading to anti-Twomey effect in warm clouds. However, further theoretical studies and in-situ observations have to be carried out to establish this effect in warm clouds as satellite retrieved AI and CDNC are subjected to retrieval uncertainties. Variations in N_d_ affect micro and macrophysical properties of clouds like cloud LWP and cloud fraction^4^. Since LWP response to aerosol perturbation depends on multiple factors like cloud geometry and thermodynamic conditions, it is quite difficult to observationally constrain aerosol induced modification on cloud LWP.

Previous studies^14^ suggested that N_d_-LWP relationship can potentially constrain aerosol impact on cloud LWP from other factors as N_d_ is largely influenced by CCN concentration and in-cloud updraft rather than relative humidity. First and second indirect effects can lead to a strong positive relationship between N_d_ and LWP; while entrainment of dry air leads^13^ to reduction in LWP with an increase in N_d_. There are also observational evidences^15^ suggesting a weak negative response of cloud LWP to N_d_ where causality is constrained; while the recent study also suggests the existence of non-linear relationships between N_d_ and LWP with an increase (decrease) in LWP for lower (higher) values of N_d_^14^. To constrain the aerosol impact on cloud LWP over the northern Indian Ocean we analysed the response of cloud LWP as a function of N_d_ and a negative relationship ($\lambda = \frac{\partial \text{ln}lwp}{\partial \text{ln}Nd}\cong -0.36$ ) is observed (Fig. 2b). Variation of LWP with N_d_ under different meteorological conditions (supplementary Figure-08) also revealed a negative relationship and rate of decrease of LWP is found to be high under unstable and moist environmental conditions. Modelling studies have demonstrated that the presence of absorbing aerosols within the non-precipitating stratocumulus clouds can reduce cloud water content leading to positive semi-direct effect^9^. The $\lambda$ observed over the study region is quite comparable to those reported^14,15^ over heavily polluted regions like ship tracks and oil refineries, where an offset of cooling is observed in comparison with the first indirect effect. Since uncertainty associated with the retrieval of N_d_ is quite high^44^, further investigations have to be carried out to quantify the aerosol induced reduction in cloud LWP over the study region.

Our analysis using satellite data revealed that enhanced presence of anthropogenic aerosols over the northern Indian Ocean during dry months modulate the microphysical properties of warm clouds which in-turn affects the regional energy budget through their direct and indirect effects. To estimate indirect radiative effect (see “Methods”), we used simultaneous quasi-collocated CERES Short Wave (SW) flux data along with physically interacting aerosol and cloud data. Variation of CERES SW flux with aerosol loading for three different conditions corresponding to (i) clear sky aerosol direct effect (Cloud Fraction = 0), (ii) low LWP clouds and (iii) high LWP clouds are shown in Fig. 2c. An increase in short wave flux can be seen under clear sky condition [estimated aerosol direct radiative effect (refer “Methods”) =  ~ − (10–12 W m^−2^)] as well as in case of high LWP clouds with an increase in aerosol loading, while a slight decrease is observed in case of low LWP clouds. Estimated intrinsic aerosol-cloud radiative forcing in the case low and high LWP clouds over the northern Indian Ocean is found to be ~ 0.95 and ~ − 0.45 W m^−2^ respectively. Our analysis suggests that over the northern Indian Ocean, aerosol interactions on warm clouds having low LWP induce a regional warming effect. Similar warming effect is also reported by other investigators for low LWP clouds under unstable and dry environments^13,45^.

Recent study^22^ using in-situ observation reported a decrease in turbulent kinetic energy (TKE) leading to an increase in atmospheric stability over the northern Indian Ocean during dry months due to enhancement in absorbing aerosol loading. Such polluted conditions suppress convective activity and also there will be a decrease in turbulent diffusion in the planetary boundary layer. As a consequence, there will be an enhancement in moisture content within the lower atmosphere leading to the formation of low-level clouds/fog. A positive relation between AOD and surface relative humidity (RH_surf_) in case of clouds with low LWP (supplementary Figure-09) and the enhancement in low-level clouds^46^ over study region can be considered as a consequence of above feedback processes. This is a self-amplifying process which will further enhance aerosol concentration within the lower atmosphere, leading to persistent low visibility fog events. Depending on cloud geometric scale and thermodynamical conditions modelling studies suggest the existence of an optimal concentration (N_op_) which is associated with maximum liquid water mass^47^. When aerosol concentration is lower than N_op_ an increase in cloud LWP is observed; while the increase in aerosol concentrations above N_op_ results in a decrease in cloud LWP (cloud suppression) due to enhanced entrainment and evaporation^48^. Analysis of Fig. 2c revealed that in case of low LWP clouds, LWP initially increased with aerosol loading and it showed a decreasing trend as AI increased beyond 0.2 (which is also reflected in the variation of upwelling SW flux with AI). This observation qualitatively infers the existence of N_op_ (quantified in terms of the AI) over the study region; however further comprehensive in-situ observations need to be carried out to quantify it.

Uncertainties associated with satellite estimation of ACI are quite high, which mainly arises from using AOD/AI as a proxy for CCN^49^. Albeit these inherent uncertainties constrain the quantification of the underlying physical processes and the associated feedback mechanism, our findings provide ample evidence for further comprehensive investigations on ACI over the northern Indian Ocean.

## Methods

MODIS sensor, on-board Terra (equator crossing time is ∼ 10:30 LT) and Aqua (equator crossing time is ∼ 13:30 LT) satellites is one of the key instruments widely used by scientific communities for studying the impact of aerosol and cloud on earth radiation budget. It has 36 spectral bands (0.4–14.4 μm) with a spatial resolution of 250 m (bands 1–2), 500 m (bands 3–7) and 1 km (bands 8–36) respectively. Sensor related specifications and detailed aerosol/cloud retrieval algorithms (collection-6) can be found elsewhere^50,51^. In the present study, we used long term (2003–2016) Level 3 Aqua MODIS aerosol and cloud properties data for studying aerosol interactions on warm clouds over the northern Indian Ocean. Aerosol and cloud data, which are co-located in space and time are only used in the analysis. Domain averages of AOD and cloud data over study regions were not done prior to the estimation of ACI as sufficient samples will not be available for estimation of ACI for different LWP ranges. Non precipitating warm marine clouds are identified from MODIS data^40^ based on following screening criteria: (a) Data corresponding to CTP less than 700 hPa are excluded to deal with shallow clouds^40^; (b) Cloud optical thickness (COT) smaller than 4 and CER less than 4 μm are excluded as there exist retrieval uncertainty^51^; (c) CER greater than 28 μm were also discarded to avoid heavy precipitating clouds^52^; (d) AOD greater than 0.7 and less than 0.05 are excluded from analysis. AOD less than 0.05 are excluded as their exist retrieval uncertainty^50^; while AOD greater than 0.7 are screened out to reduce uncertainties associated with cloud adjacent AOD retrievals (partially cloudy effect and swelling effect etc.)^53^. Frequency distribution of AOD over the study region (supplementary Figure-03) revealed that usage of criteria (d) includes 99% of all AOD data over SA1 and 90% at SA2.

Aerosol Cloud Interaction (ACI) is quantified for a fixed LWP is estimated as^54^:

1 $$ACI=\frac{-\partial lnCER}{\partial lnAI}{|}_{LWP}$$

where, Aerosol Index (AI) is the product of AOD and AE is widely used as a proxy for CCN concentration in ACI estimation studies^55^. Student t-test is performed between AI and CER for each LWP bin prior to ACI estimation and we found a statistically significant correlation (p < 0.05) between them (supplementary Table-02).

CALIPSO^56^ is the only satellite currently in orbit that provides the vertical distribution of clouds and aerosols. In this study, we used long term (2006–2016) vertical layer information’s of aerosol and clouds over the northern Indian Ocean. We analysed ~ 4200 CALIPSO tracks for this study. Only those profiles are considered where aerosol particles and cloud layers are physically interacting i.e. aerosol bottom and cloud top layer is within 100 m range^40^. However, a recent observational study^57^ reported that ACI estimated using above-cloud aerosol, even within 100 m of cloud top, may not accurately reflect the relationship. To reduce the uncertainty to an extent, profiles having mono aerosol and cloud layer are only used in the analysis. Geolocations of physically interacting aerosol and cloud layers over the northern Indian Ocean during dry months can be found in supplementary Figure-07a. Further, Aqua MODIS retrieved Level-2 aerosol and cloud optical properties are averaged within 50 km radius from CALIOP target and the screening criteria for identifying warm clouds are applied prior to analysis. Methodology elucidating multi-satellite approach for estimating ACI can be found elsewhere^27,40^ and details of data set used in multi-satellite estimation of ACI are shown in Supplementary Table-04.

Temperature and humidity profiles retrieved from Atmospheric Infrared Sounder (AIRS) are used to estimate LTS and FTH. LTS is defined as the potential temperature (θ_p_) difference between 700 hPa and mean surface level^58^ and relative humidity at 700 hPa is considered as FTH.

To estimate CDNC (N_d_, cm^−3^) we used Level-2 collection 6 MODIS cloud product data set. For an adiabatic cloud CDNC is related to CER and COT (τ_c_) as follows^59^:

$${N}_{d}=\Gamma \frac{{10}^{1/2}}{4\pi {\rho }_{w}^{1/2} k}\frac{{\tau }^{1/2}}{{r}^{5/2}} \sim \;= 1.4067*10^{-6}* \frac{{{\tau }_{c}}^\frac{1}{2}}{{r}^\frac{5}{2}} \left[{\text{If}\; \text{N}_\text{d} \;\text{is}\; \text{assumed} \; \text{to} \; \text{be} \; \text{constant}\; \text{with} \; \text{height}}\right]$$

In order to ensure only valid cloud observations for the estimation of CDNC, we used following set of screening criteria:

- Cloud phases retrieved by MODIS infrared and visible channels should indicate liquid phase^59^.
- MODIS estimated CER at 3 different wavelengths (3.7, 2.1 and 1.6 µm) should be in descending order (i.e. CER_3.7_ > CER_2.1_ > CER_1.6_). This criterion ensures the fundamental assumptions (adiabatic and homogenous clouds) associated with CDNC retrievals^59^.
- Cloud top temperature should be between 268 and 300 K to ensure the selection of warm clouds pixels only.
- Cloud optical depths smaller than 4 and CER smaller than 4 µm are not considered due to high retrieval uncertainty^51^.
- Only pixels with cloud fraction greater than 0.9 are considered for analysis and pixels with inhomogeneity index (Cloud_Mask_SPI) greater than 30 are excluded. Inclusion of this criteria helps to avert the uncertainties associated with partially cloudy/broken cloud pixels in CDNC retrieval^14^.
- Only pixels with solar zenith angle less than 65° and sensor zenith angle less than 41.4° are only used in the analysis^44^. Usage of this criterion minimises the uncertainties associated with satellite viewing geometry in CDNC retrieval.

Aerosol indirect radiative effect is estimated by following^60^

2 $$\frac{\partial {C}_{sw}}{\partial \text{ln}(AI)}=[\stackrel{-}{{C}_{m}}\left(\frac{\partial {A}_{clr}}{\partial \text{ln}\left(AI\right)}-\frac{\partial {A}_{cld}}{\partial \text{ln}\left(AI\right)}\right)+ \overline{{A}_{clr}-{A}_{cld}}\frac{\partial {C}_{f}}{\partial \text{ln}(AI)}]{\overline{F}}^{\downarrow }$$

where, C_sw_ is the quasi coincident CERES short wave flux at Top Of the Atmosphere (TOA), C_f_ is the cloud fraction, ${A}_{clr}$ is the clear sky albedo, ${A}_{cld}$ is the cloudy sky albedo (${A}_{cld}=\left[{A}_{all}-\left(1-{C}_{f}\right){A}_{clr}\right]/{C}_{f}$), $\overline{{C}_{m}}$ is the mean marine warm cloud coverage (~ 31%) and ${\overline{F}}^{\downarrow }$ is the incoming solar radiation. First-term on the right-hand side of Eq. (2) determines that effect of changes in cloud microphysics due to aerosol loading on SW flux at TOA (intrinsic radiative effect), and the second term denotes the effect of changes in cloud macrophysics due to aerosol loading on SW flux at TOA (extrinsic radiative effect).

Shortwave Aerosol Direct Radiative Effect (ADRE) is estimated using simultaneous daily MODIS aerosol, cloud and CERES SSF SW flux data. To estimate ADRE only those pixels are selected where MODIS Cloud Fraction is zero. ADRE is estimated by following^61^ as

3 $${\text{ADRE}}_{{{\text{TOA}}}} = {\text{F}}_{{{\text{clr}}}} - {\text{F}}_{{{\text{aero}}}}$$

where, F_clr_ is considered as the SW flux at TOA for minimum AOD condition. In the present study, we estimated F_clr_ by interpolating the regression relation between AOD and SW flux for zero AOD condition (Estimated F_clr_ is ~ 78 W m^−2^).

## Supplementary information

Supplementary Information.

## Data Availability

MODIS data are available from the NASA Goddard Space Flight Center (‘https://ladsweb.modaps.eosdis.nasa.gov/’). CERES data used in this study are obtained from the NASA Langley Research Center, Atmospheric Science Data Center (https://ceres.larc.nasa.gov/). AIRS data can be obtained from, (‘https://airs.jpl.nasa.gov/data/get_data’). CALIPSO data are available online from https://www-calipso.larc.nasa.gov/.

## Code availability

MATLAB codes used in this study are available upon request to the corresponding author.
