# Supplementary material for: Anthropogenic emissions from South Asia reverses the aerosol indirect effect over the northern Indian Ocean
Source: Sci Rep. 2020 Oct 27;10:18360. doi: 10.1038/s41598-020-74897-x (PMC7591568; doi:10.1038/s41598-020-74897-x)
Supplement: Supplementary file 1 — Supplementary Information. [file 41598_2020_74897_MOESM1_ESM.docx]

**Anthropogenic Emissions from South Asia reverses the aerosol Indirect Effect over the northern Indian Ocean**

**Subin Jose*, Vijayakumar S. Nair and S. Suresh Babu**

Space Physics Laboratory, Vikram Sarabhai Space Centre, Trivandrum

*email: [**subinjose22@gmail.com**](mailto:subinjose22@gmail.com)

**Supplementary analysis, tables and figures**

**Trend analysis**

AOD retrieved from passive remote sensing polar satellites like MODerate resolution Imaging Spectro-radiometer (MODIS), Multi Imaging Spectro Radiometer (MISR), Advanced Very High Resolution Radiometer (AVHRR) and Sea-viewing Wide Field-of-view Sensor (SeaWiFs) are utilized to study the long term trend over the study area. In the present study we used monthly mean Level -3 data from respective satellites for analysis. Specification of each sensors and their respective uncertainties in AOD retrieval can be found else where^1–5^.

Trend in columnar aerosol loading and its statistical significance is estimated by using a trend model ^6^ as

$y\left( t \right)=\mu+\omega x\left( t \right)+\varepsilon\left( t \right)$ (1)

where y(t) is the geophysical variable as a function of time, t; μ is an offset term; ω is the trend per year; x(t) is the independent variable represented by time in this study and ε(t) is the noise (i.e. residuals on the straight-line fit) in the time series which is assumed to be auto-regressive of the order of 1. Significance of the trend is assessed using the ratio of the absolute trend to its uncertainty (|ω/σ_ω_|), where uncertainty in trend (σ_ω_) is calculated as

$\sigma_{\omega}=\frac{\sigma_{\epsilon}}{N^{3/2}}\sqrt{\frac{1+\varphi}{1-\varphi}}$ (2)

where σ_ε_ is the standard deviation of the noise (ε) and N is the total number of years and φ is the is the autocorrelation coefficient. Trend is considered significant at a 5% significance level or 95% confidence level when this ratio (|ω/σ_ω_|) is greater than 2.

**Estimation of anthropogenic fraction**

Anthropogenic fraction of total AOD over SA1 and SA2 is estimated from daily Level 3 MODIS satellite following ^7^. Anthropogenic AOD (τ_anth_) is estimated as:

$\tau_{anth}= \frac{\left( f_{550}-f_{dust} \right)\tau_{550}-(f_{marine}-f_{dust})\tau_{marine}}{f_{anth}-f_{dust}}$ (3)

Where f_550_ is bounded by f_anth_ ≥ f_550_ ≥ min{f_anth_, f_dust_}. f_550_ is fine mode fraction estimated as the ratio of fine mode AOD and total AOD (τ_550_). The f_anth_, f_dust_, and f_marine_ are the fine mode fraction thatcorresponds to the anthropogenic, dust, and maritime aerosols, respectively. We used a priori information onthe fine mode fraction of these aerosol types as follows f_anth_ = 0.889, f_dus_ = 0.309 and f_marine_ = 0.475 following ^8^.

Supplementary Figure -01





Figure S1: Long term variation in AOD retrieved from various satellites over SA1 and SA2.

Supplementary Figure- 02


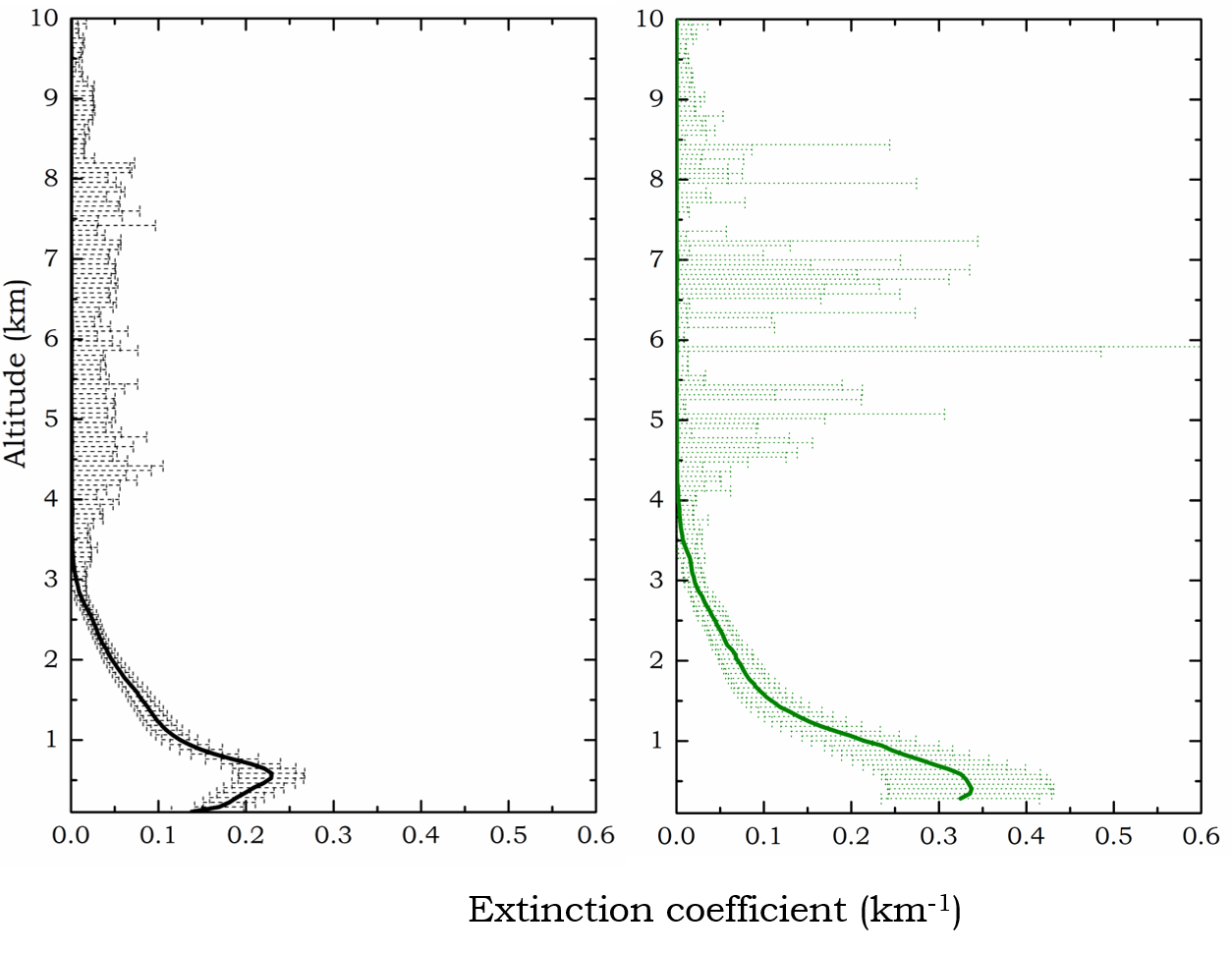


Figure S2- Mean vertical profile of aerosol extinction coefficient over SA1 and SA2 retrieved from CALIPSO.

Supplementary Figure- 03





Figure S3- Frequency distribution of aerosol (AOD, Angstrom Exponent), cloud parameters (CER, COT, LWP, CF, CTP) and precipitable water vapour over SA1 and SA2.

Supplementary Figure- 04


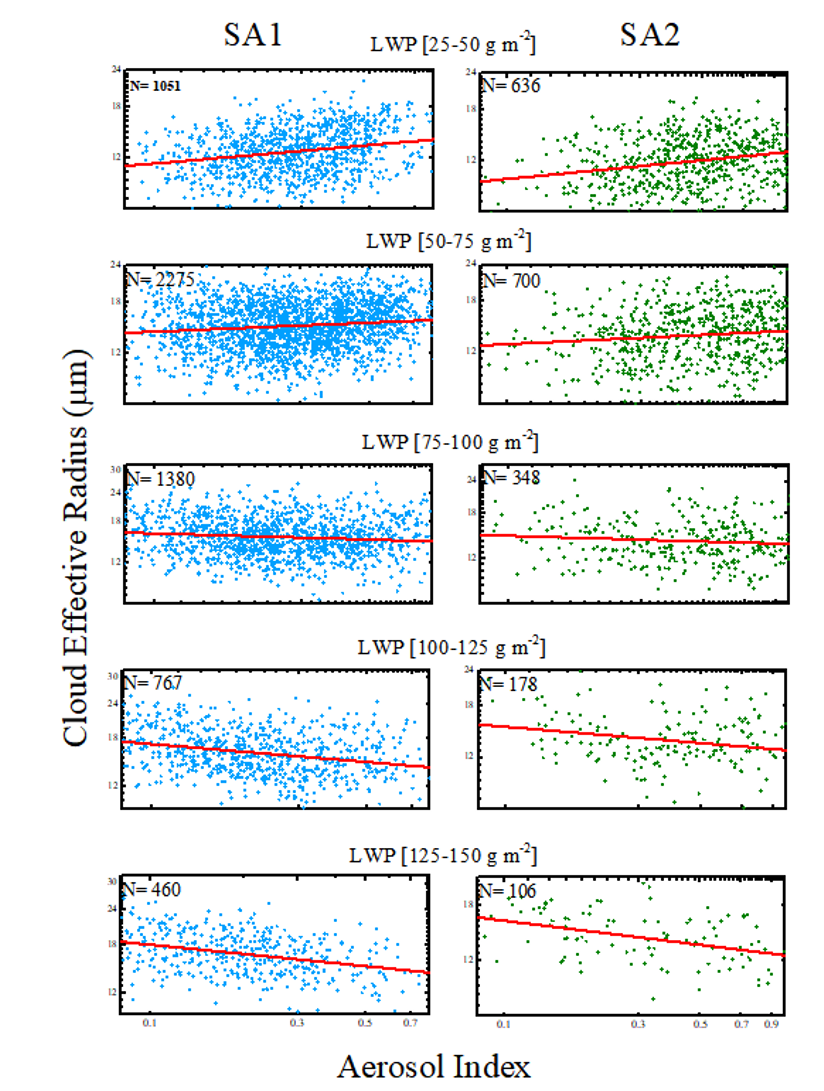


Figure S4: CER as a function of aerosol index (log-log scale) for different LWP over SA1 and SA2

Supplementary Figure- 05


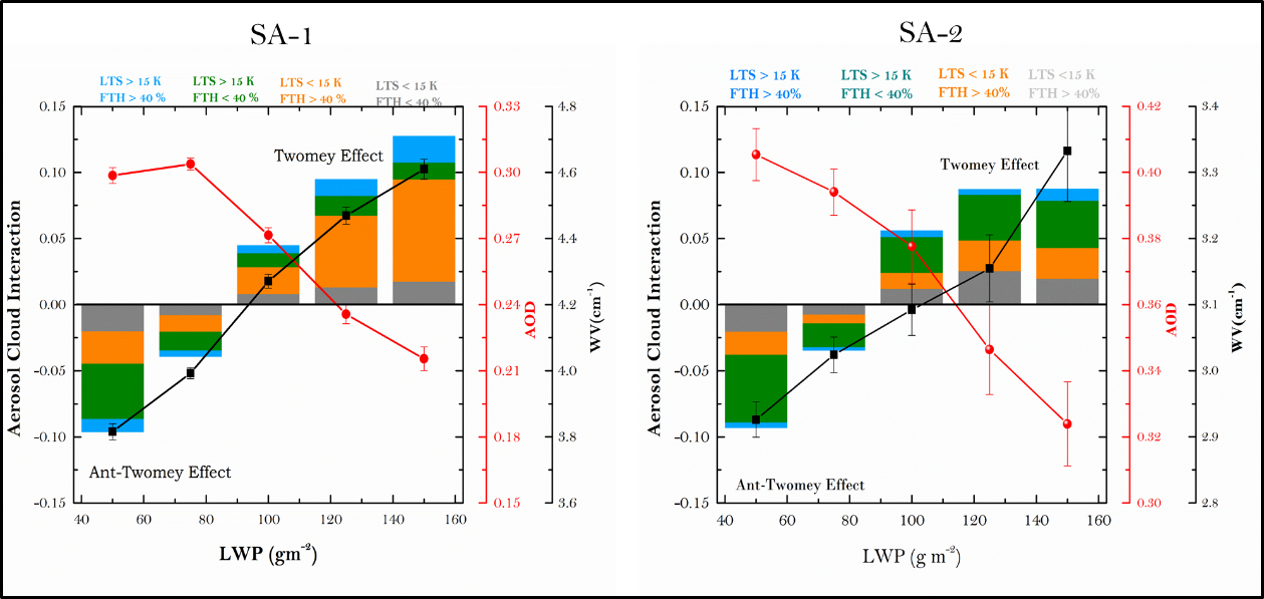


Figure S5: Aerosol cloud interaction as a function of cloud liquid water path for different LTS and FTH conditions over SA1 and SA2. AOD and water vapour corresponding to each LWP bins are represented by red and black curve respectively

Supplementary Figure- 06


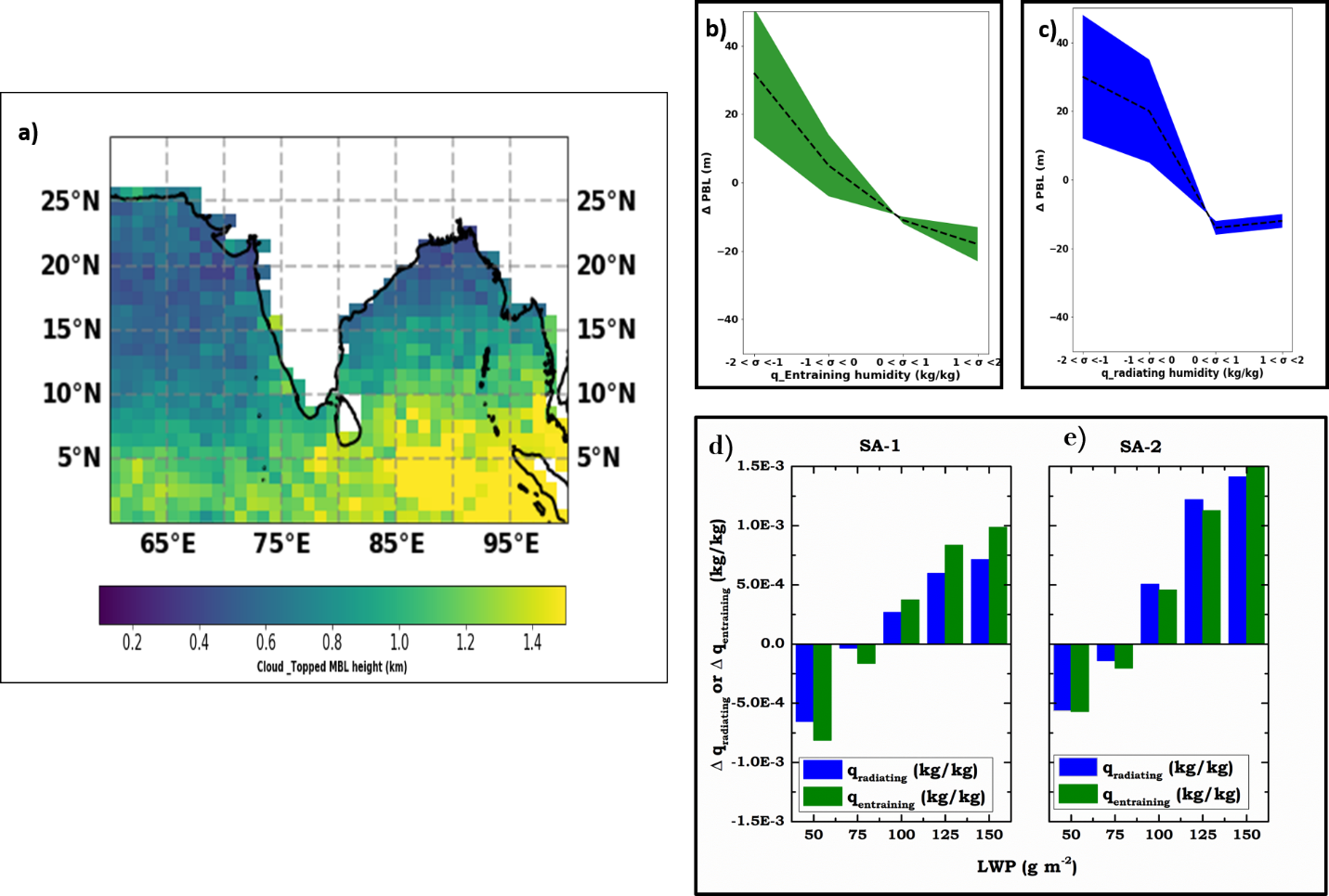


Figure S6 a): Mean cloud-topped planetary boundary layer height (km) over the northern Indian Ocean during winter months. Variation of cloud-topped planetary boundary layer height anomaly (m) as a function of Entraining humidity (b) and radiating humidity (c). Shaded portion indicates the standard deviation of MBL anomaly. Anomaly in radiating and entraining humidity as a function of cloud water content over (d) SA1 and (e) SA2.

Supplementary Figure- 07


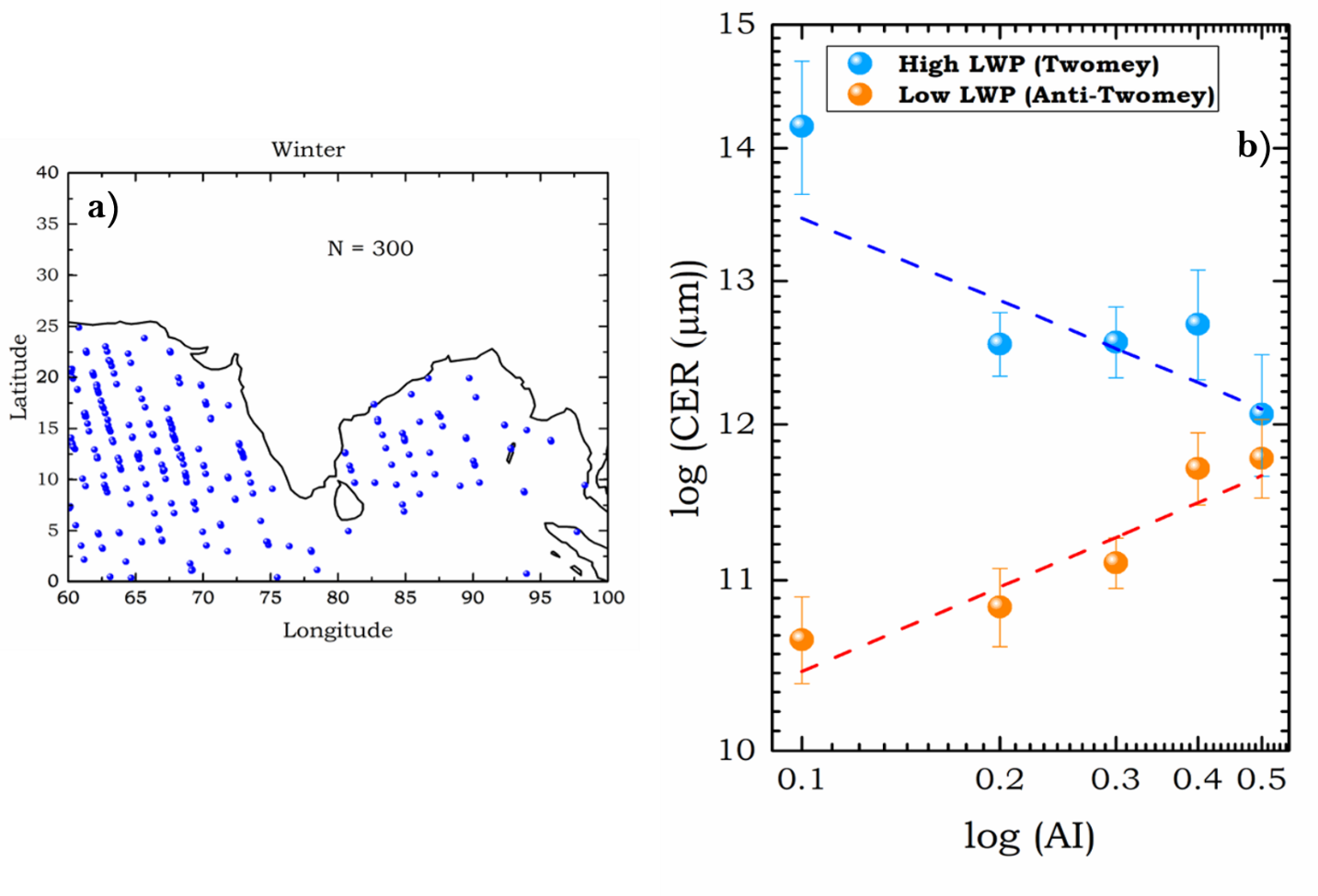


Figure S7- a) Geolocation of physically interacting aerosol and cloud layers as observed by CALIPSO during winter months over NIO. b) Variation of CER as function of aerosol index in the case of physically interacting aerosols and clouds.

Supplementary Figure – 08


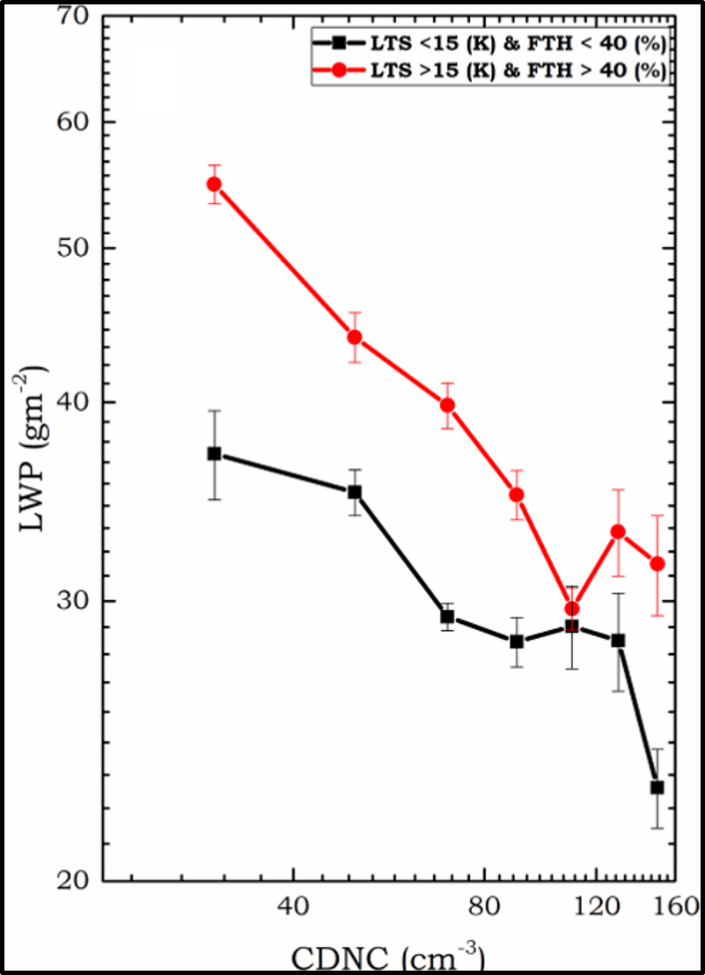


Figure S8: Cloud LWP as a function of CDNC under different meteorological conditions.

Supplementary Figure – 09





Figure S9: Variation of surface humidity normalised by aerosol loading for different LWP bins.

Supplementary Table -01

Table1: Statistics associated with multi-satellite AOD trend analysis over SA1 and SA2

| Satellite | Period | λ (µm) | Study area | Mean±Std | Trend/yr(Sig) |
| --- | --- | --- | --- | --- | --- |
| AVHRR | 1981-2015 | 0.63 | SA1 | 0.2±0.005 | 0.003(10.4) |
|  |  |  | SA2 | 0.26±0.07 | 0.005(8.6) |
| SeaWiFS | 1998-2010 | 0.55 | SA1 | 0.2±0.02 | 0.005(8.4) |
|  |  |  | SA2 | 0.31±0.06 | 0.014(8.3) |
| MODIS-  Terra | 2001-2017 | 0.55 | SA1 | 0.3±0.04 | 0.007(2.1) |
|  |  |  | SA2 | 0.4±0.07 | 0.011(12) |
| MODIS-  Aqua | 2003-2017 | 0.55 | SA1 | 0.28±0.04 | 0.007(2.3) |
|  |  |  | SA2 | 0.39±0.05 | 0.009(10) |
| MISR | 2001-2017 | 0.55 | SA1 | 0.27±0.02 | 0.0035(5.4) |
|  |  |  | SA2 | 0.36±0.04 | 0.006(8.2) |

Supplementary Table -02

Table2- Statistics associated with estimation of ACI over SA1 and SA2. Terms in the parenthesis include number of data points, correlation coefficient and significance of correlation.

| LWP bin (gm^-2^) | $-\frac{\partial ln(CER)}{\partial ln(AI)}$  Over SA1 | $-\frac{\partial ln(CER)}{\partial ln(AI)}$  Over SA2 | LTS±STD (K)  Over SA1 | LTS±STD (K)  Over SA2 |
| --- | --- | --- | --- | --- |
| 25 ≤ LWP ≤ 50 | -0.097  (1038,0.24,1.73E-15) | -0.093  (529,0.21,8.6E-7) | 15.14±0.035 | 15.45±0.09 |
| 50 ≤ LWP ≤ 75 | -0.04  (2346,0.11,2.86E-8) | -0.035  (576,0.1,0.006) | 15.03±0.02 | 15.36±0.08 |
| 75 ≤ LWP ≤ 100 | 0.045  (1419,-0.13,4.9E-7) | 0.06  (301,-0.2,0.008) | 14.75±0.023 | 15.26±0.1 |
| 100 ≤ LWP ≤ 125 | 0.0951  (794,-0.3,1.3E-16) | 0.09  (162,-0.25,0.001) | 14.6±0.03 | 14.82±0.1 |
| 125 ≤ LWP ≤ 150 | 0.13  (466,-0.39,1E-18) | 0.09  (98,-0.313,0.0017) | 14.6±0.04 | 15.03±0.1 |

Supplementary Table -03

Table 3- Previous studies on ACI over NIO

| **Studies** | **Platform** | **Observation** |
| --- | --- | --- |
| Chylek et al., 2006^9^ | Satellite [MODIS] | Twomey Effect |
| Sekiguchi et al., 2003^10^ | Satellite [AVHRR, MODIS] | Weak relation between CER and CCN |
| Myhre et al., 2007^11^ | MODEL, Satellite | Twomey effect |
| Bulgin et al., 2008^12^ | Satellit, ATSR-2 | Anti-Twomey effect |
| Jones and Christrofer., 2008^13^ | Satellite | Anti-Towmey effect [LWP < 45 gm^-2^] |
| Chen et al., 2014^14^ | Multi satellite | Twomey effect (Northern hemisphere) |

Supplementary Table -04

Table 4. Level 2 MODIS, CALIOPSO products used to characterize aerosol and cloud properties.

| Satellite | | Products | Resolution |
| --- | --- | --- | --- |
| MODIS | Aerosol (MYD04 Level 2 Collection 6) | Aerosol_Optical_Depth_Average_Ocean_Mean | 10 km |
|  | Cloud (MYD06 Level 2 Collection 6) | Cloud_Effective_Radius_Liquid_Mean  Cloud_Water_Path_Liquid_Mean  Cloud_Optical_Thickness_Liquid_Mean  Cloud_Fraction_Day_Mean  Cloud_Top_Pressure_Day_Mean | 1 km  1 km  1 km  5 km  5 km |
| CALIPSO | Aerosol (05kmALay) | Layer_Top_Altitude  Layer _Base_Altitude | 5 km  5 km |
|  | Cloud(05kmCLay) | Layer_Top_Altitude  Layer _Base_Altitude | 5 km  5 km |
| CERES | SSF-Level 2 Ed-4 | Short wave Flux | 20 km |

References

1. Zhao, T. X. P. *et al.* Study of long-term trend in aerosol optical thickness observed from operational AVHRR satellite instrument. *J. Geophys. Res. Atmos.***113**, D07201 (2008).

2. Sayer, A. M. *et al.* SeaWiFS Ocean Aerosol Retrieval (SOAR): Algorithm, validation, and comparison with other data sets. *J. Geophys. Res. Atmos.***117**, n/a-n/a (2012).

3. Martonchik, J. V, Kahn, R. A. & Diner, D. J. Retrieval of aerosol properties over land using MISR observations. in *Satellite aerosol remote sensing over land* 267–293 (Springer, 2009).

4. Deschamps, P.-Y. *et al.* The POLDER mission: Instrument characteristics and scientific objectives. *IEEE Trans. Geosci. Remote Sens.***32**, 598–615 (1994).

5. Levy, R. C. *et al.* The Collection 6 MODIS aerosol products over land and ocean. *Atmos. Meas. Tech.***6**, 2989–3034 (2013).

6. Weatherhead, E. C. *et al.* Factors affecting the detection of trends: Statistical considerations and applications to environmental data. *J. Geophys. Res. Atmos.***103**, 17149–17161 (1998).

7. Kaufman, Y. J. *et al.* Aerosol anthropogenic component estimated from satellite data. *Geophys. Res. Lett.***32**, (2005).

8. Lakshmi, N. B., Nair, V. S. & Suresh Babu, S. Vertical Structure of Aerosols and Mineral Dust Over the Bay of Bengal From Multisatellite Observations. *J. Geophys. Res. Atmos.* (2017). doi:10.1002/2017JD027643

9. Chylek, P. *et al.* Aerosol indirect effect over the Indian Ocean. *Geophys. Res. Lett.***33**, L06806 (2006).

10. Sekiguchi, M. A study of the direct and indirect effects of aerosols using global satellite data sets of aerosol and cloud parameters. *J. Geophys. Res.***108**, 4699 (2003).

11. Myhre, G. *et al.* Comparison of the radiative properties and direct radiative effect of aerosols from a global aerosol model and remote sensing data over ocean. *Tellus, Ser. B Chem. Phys. Meteorol.* (2007). doi:10.1111/j.1600-0889.2006.00238.x

12. Bulgin, C. E. *et al.* Regional and seasonal variations of the Twomey indirect effect as observed by the ATSR-2 satellite instrument. *Geophys. Res. Lett.***35**, (2008).

13. Jones, T. A. & Christopher, S. A. Seasonal variation in satellite-derived effects of aerosols on clouds in the Arabian Sea. *J. Geophys. Res. Atmos.***113**, D09207 (2008).

14. Chen, Y. C., Christensen, M. W., Stephens, G. L. & Seinfeld, J. H. Satellite-based estimate of global aerosol-cloud radiative forcing by marine warm clouds. *Nat. Geosci.***7**, 643–646 (2014).
